# Determination and ecological risk assessment of two endocrine disruptors from River Buffalo, South Africa

**DOI:** 10.1007/s10661-020-08717-0

**Published:** 2020-11-06

**Authors:** Lamidi W. B. Olaniyan, Anthony I. Okoh

**Affiliations:** 1 SAMRC Microbial Water Quality Monitoring Centre, University of Fort Hare, Alice, 5700 South Africa; 2 Applied and Environmental Microbiology Research Group (AEMREG), Department of Biochemistry and Microbiology, University of Fort Hare, Alice, 5700 South Africa; 3 Present Address: Biochemistry Department, Faculty of Basic Medical Sciences, Ladoke Akintola University of Technology Ogbomoso, Ogbomoso, Nigeria

**Keywords:** Alkylphenol, Ecotoxicity, Estrogenicity, Hazard quotient, Octylphenol, Triclosan

## Abstract

4-tert-Octylphenol (4-tOP) and triclosan (TCS) are endocrine disruptors which have been detected in environmental matrices such as air, soil and water at ultra-low levels. Exposure to endocrine disruptors may account at least in part, for the global increase in the incidence of non-communicable diseases like cancers and diabetes and may also lead to an imbalance in the aquatic ecosystem. River Buffalo is an important natural resource in the Eastern Cape of South Africa serving more than half a million people. The presence of the two compounds in the river water hitherto unknown was investigated during winter seasons using solid-phase extraction and gas chromatography–mass spectrometric techniques. The sampling points differed by some physicochemical parameters. The concentration of 4-tOP ranged 0–755 ng/L, median value 88.1 ng/L while that of TCS ranged 0–1264.2 ng/L and the median value was 82.1 ng/L. Hazard quotient as an index of exposure risk varied according to daphnids ˃ fish ˃ algae for 4-tOP exposure while HQ for TCS exposure was algae > daphnids = fish showing that both compounds were capable of causing imbalance in the aquatic ecosystem.

**Figure:**
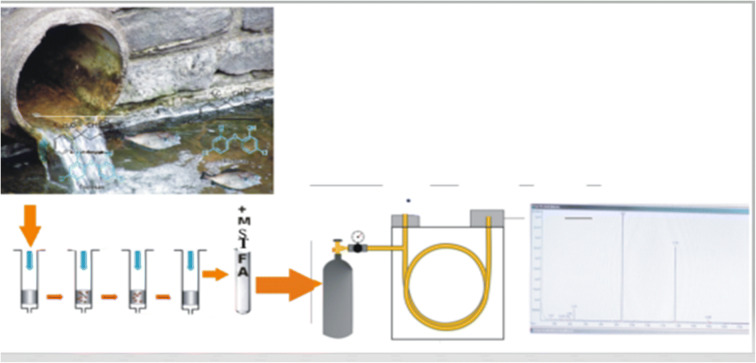
Graphical abstract

Graphical abstract

## Introduction

Humans beyond doubt are exposed to numerous chemicals from different routes including contaminated water and personal care products. Some of these chemicals to which humans and wildlife are frequently exposed from nearly all environmental matrices have been shown to interfere with endocrine system (Careghini et al. 2015). These endocrine disruptors (ED) are of various classes including drugs, industrial chemicals, household items and personal care products where they ultimately find their ways to the environmental matrices such as surface and underground waters (Gore et al. 2015) via urban and industrial runoffs (Water Research Commission 1996). Following their frequent detection in environmental matrices such as air (Mandin et al. 2016), water (reviewed by Olaniyan et al. 2018) and soil (Butler et al. 2012) though at ultra-trace (ng/L) levels, their adverse health effects and negative environmental impacts have become worrisome (Dupuis et al. 2012; Barber et al. 2015; Kong et al. 2015). Their adverse effects leading to certain forms of cancers and other non-communicable diseases such as diabetes in man (Diamanti-Kandarakis et al. 2009; Lyche et al. 2011) and adverse effect on the population of aquatic lives (Jobling et al. 2002; Diao et al. 2017) have been reported. The exposure of zebrafish larvae to ED such as triclosan at environmentally relevant concentrations delayed metamorphosis, impaired fecundity and fertility presumed to be related to altered thyroid hormone homeostasis (Stenzel et al. 2019).

**Fig. 1:**
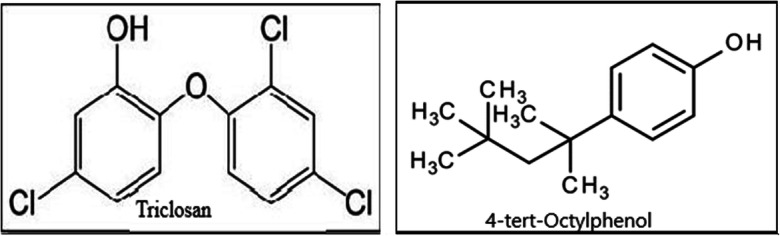
Chemical structures of TCS and 4-tOP

4-tert-Octylphenol or 4- (1, 1, 3, 3-tetramethylbutyl) phenol (4-tOP) (Fig. 1) and triclosan, (5-chloro-2-(2,4-di chlorophenoxy) phenol) (TCS) are phenolic chemicals of high production volume (˃ 1000 t/year) worldwide (Brooke et al. 2005). They have both been reported for endocrine interference among other adverse effects on human and wildlife (Paris et al. 2002; Shim et al. 2016; Sheikh 2020). 4-tOP is an alkylphenol and a degradation product of non-ionic detergent alkylphenol polyethoxylates (Kovarova et al. 2013). TCS is a synthetic broad-spectrum antimicrobial agent present in some personal care products (MacIsaac et al. 2014). The chemical compounds ultimately find their way to water resources (Lee et al. 2013). An aquatic ecosystem is a multi-stressed environment and chemicals coexisting in the aquatic milieu can act cumulatively and cause health problems even in humans (Rochester 2013; Evans et al. 2016). In South Africa, the poor state of the health of aquatic ecosystems has provoked public attention in recent past (Dalvie et al. 2003; Bollmohr et al. 2009) and a national programme has been launched aimed “to promote standardised and continuous monitoring and to provide reports on river health” in the Eastern Cape (ECRHP 2004). River Buffalo is on the east coast in the Eastern Cape Province of South Africa. It is a system of 125 km with a catchment of 1276 km^2^ (ECRHP 2004) populated by more than 570,000 inhabitants which rely on the river for their domestic, industrial and agricultural needs (ECRHP 2004). The river is being impacted by wastewater effluents containing domestic and industrial wastes, empties into the Indian Ocean in the city of East London (ECRHP 2004). So far, no study has been dedicated to the detection of 4-tOP and TCS in the Buffalo River; therefore, the exposure impacts on humans and aquatic wildlife are unknown. Physicochemical parameters which vary according to season are universally acknowledged as an important index of surface water quality (Singh and Sharma 2016). The activities peak in winters or during dry seasons when rivers normally experience low flow as the result of reduced rainfall (Lee et al. 2013). The concentration of chemical pollutants is therefore expectedly high following reduced dilution (Wu et al. 2013). Thus, this investigation was carried out during the winter periods.

## Materials and methods

### Description of study areas

**Fig. 2:**
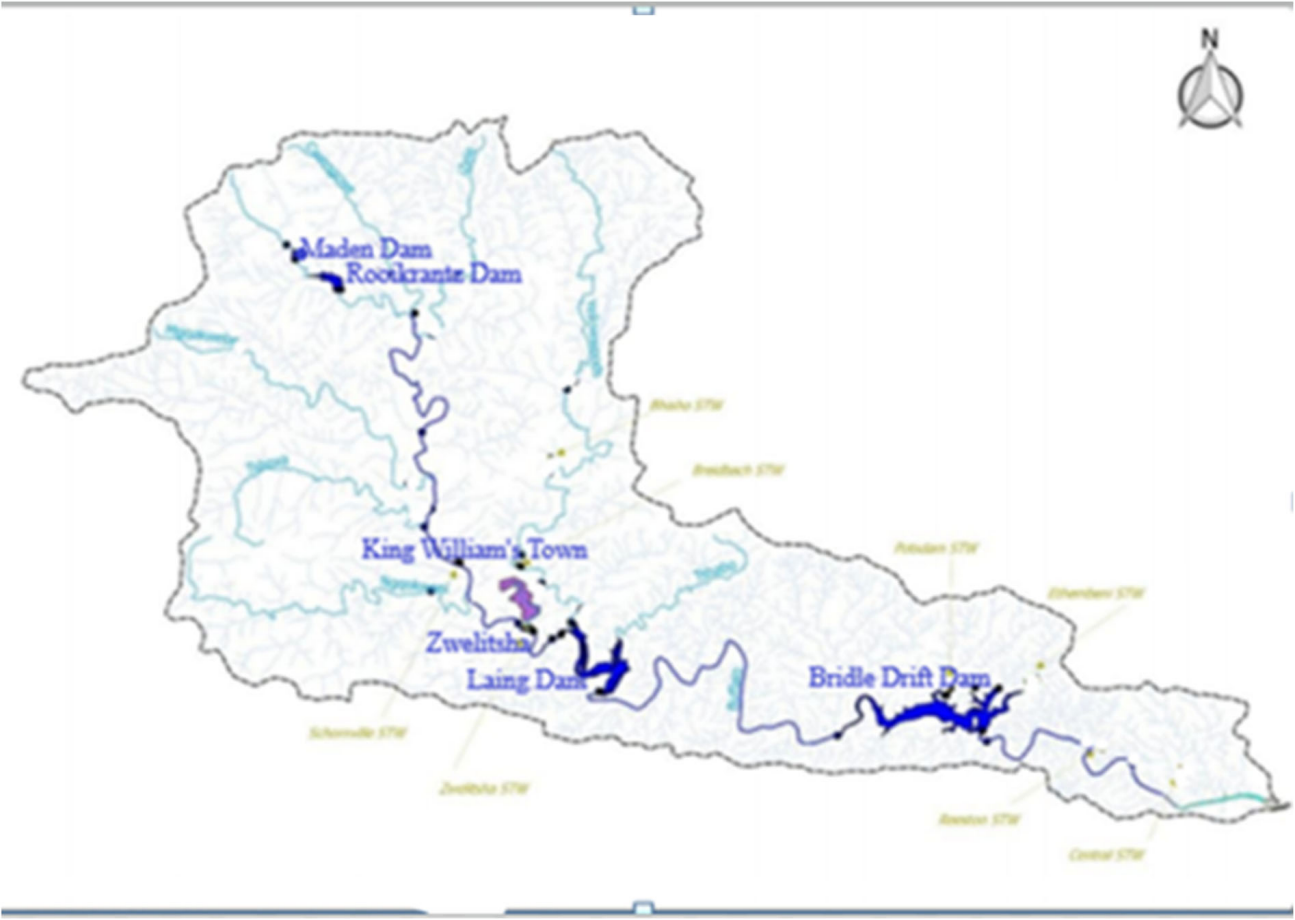
Sampling sites along the course of River Buffalo

River Buffalo takes its source from Amatole Mountains lying between King Williams Town and Stutterheim. The river passes through Zwelitsha and Mdantsane and through low altitude coastal forest in the lower reaches from Bridle Drift Dam to the head of the estuary in East London (Fig. 2; O’Keeffe et al. 1996). The major tributaries of the Buffalo River are the Mgqakwebe, Ngqokweni and Yellowwoods rivers which join the mainstream above Laing Dam (ECRHP 2004). The middle reaches are made up of the urban/industrial complex of King Williams Town/Zwelitsha to Laing dam, and an area of agricultural land downstream of Laing dam (Table 1). Apart from the tributaries (Fig. 2), Buffalo River is significantly impacted by De Gama textiles mills which contribute to the river contamination.

**Table 1:** Sampling sites description (Adapted from ECRHP, 2004)

Sites	Coordinates	Descriptions
Maden dam (MD)	32° 44′ 24′′ S 27° 17′ 57′′ E	Upstream. A near-pristine site. Minimal human-related activity, possibly impacted by riparian vegetation.
Rooikrantz dam (RD)	32° 45′ 18′ ′ S 27° 19′ 44′′ E	Four kilometres downstream of MD. Minimally impacted by human-related activities such as fishing, grazing and ploughing.
King William’s Town (KW)	32° 52′ 52′′ S 27° 22′ 54′′ E	Middle reach. An impacted site, especially by human-related activities such as domestic wastes dump, industrial wastes discharges as well as discharges from River Mgqakwebe.
Zwelitsha town (ZW)	32° 56′ 14′ ′ S 27° 27′ 57′′ E	Middle reach. Impacted by multiple disturbances associated with human activities, discharges from the tributary Ngqokweni and wastewater treatment plants.
Laing dam, a weir (LD)	32° 55′ 54′′ S 27° 28′ 22′′ E	Low reaches. Impacted by both human-related activities such as ploughing as well as discharges from the Yellowwoods River.
Bridle Drift dam (BD)	32° 58′ 35′′ S 27° 42′ 30′′ E	Downstream, 40 km east of LD. Impacted by both human-related activities such as agriculture, fishing as well as discharges from sewers.

### Chemicals

4-tOP (97%; CAS 140-66-9)) and TCS (99%; CAS 3380-34-5), N-methyl-N-(trimethylsilyl)-trifluoroacetamide (MSTFA) and terphenyl phosphate were products of Sigma-Aldrich. Anhydrous sodium sulphate and organic solvents (HPLC grade) were provided by local suppliers. Glass fibre filters (GF/F, pore size 0.45 μm) were supplied by Whatman. All the glassware used for analyses were washed, rinsed with distilled water and finally baked at 450 °C for 6 h.

### Standard solutions

Individual stock solutions of 4-tOP and TCS were prepared in acetone and were mixed from which recovery spikes and calibration solutions were prepared. All solution standards were stored in the amber-coloured bottles at − 2 °C.

### Sampling method and treatment

Sampling took place between May and July of 2016 and 2017 which were the winter periods (South African Weather Service). Composite water samples in triplicates obtained from the river just below the surface were stored in treated 1-L amber glass bottles. The sampling sites from upstream to downstream were Maden Dam (MD), Rooikrantz Dam (RD), King William’s Town (KW), Zwelitsha (ZW), Laing Dam (LD) and Bridle Drift Dam (BD) in Mdantsane; all were the river catchments (Fig. 2; Table 1). The water samples were acidified to pH 2 with 4 M HCl to prevent microbial degradation of the analytes and were placed in coolers containing ice packs while in transit to the laboratory. Approximate coordinates of the sampling sites were measured (Table 1) using global positioning systems. Sampling was undertaken monthly from May to July of 2016 and 2017, early in the mornings. The physicochemical characteristics of the samples were measured on-site using a HANNA HI 98195 multiparameter analyser and HACH turbidimeter.

### Sample extraction

Aliquots of 1 L of water samples were vacuum filtered through glass fibre filters (GF/F, pore size 0.45 μm) to retain particulate matter and other suspended solid matters. Sample extraction and analytes derivatization were carried out with slight modifications to the method of Yang et al. (2013). Solid-phase extraction (SPE) was performed with SPE manifold Supelco Visiprep™ Sep-Pack system (Milford, MA, USA) and Strata C-18 cartridge (500 mg/6 mL) from Phenomenex (Macclesfield, UK) as sorbents with an external AP-02B vacuum pump. The sorbents were conditioned by 6.0 mL each of acetone and methanol in that order to wash away the impurities, and subsequently with 6.0 mL of acidified ultra-pure water (pH 2) to activate the SPE column. The water sample was percolated through the SPE cartridges at a flow rate of 5.0 mL/min. The cartridges were air-dried and eluted the following day with 10.0 mL acetone; flow rate 1 mL/min. Baked 1.0 g anhydrous sodium sulfate (400 °C for 4 h) was added to the eluate to remove the moisture. The extracts were concentrated to approximately 1 mL using a rotary evaporator and then transferred into GC vials. Each sample extract to which 20 μL 0.2 mg/mL terphenyl phosphate was added as the internal standard was evaporated to dryness in a gentle stream of N_2_ followed by derivatization using 100 μL MSTFA incubated at 70 °C for 30 mins.

### Instrumental analysis

Analysis of the derivatized compounds was performed using a gas chromatograph 7890A (Agilent Technologies, Willmington, DE, USA) with an Agilent Technologies 7693 interfaced with a quadrupole mass spectrometer. The chromatograph with autosampler utilized helium as the carrier gas. Electron impact ionization was at 70 eV in splitless injection mode. GC transfer line to MS was 280 °C while the interface temperature was at 270 °C, and the source temperature was 230 °C. Chromatographic separation was carried out using a 30-m length, 0.32-mm internal diameter, fused silica column HP-5MS with 0.25-μm film thickness. Temperature program was held at 80 °C for 1 min to 248 °C at 15 °C/min held for 1 min then up to 280 °C at 3 °C/min, total run time was 23.9 min. The identities of the compounds were confirmed by comparison with the standards, considering full mass spectra and retention times, and also with the help of the NIST98 standard mass spectral library. External calibration was used to quantify the extract after SPE with the standard mixture. The GC was externally calibrated at six standard levels 0.5, 1, 2, 4, 8 and 10 μg/L for the compounds which were both linear (*r*^2^ > 0.99).

### Ecological impact assessment

Ecological risk assessment was carried out on the pollutant-sensitive aquatic (freshwater) organisms namely the algae (*Chlamydomonas reinhardtii*), water flea (*Daphnia magna*) and rainbow trout fish (*Oncorhynchus mykiss*). Hazard quotient (HQ), as well as individual and total estrogenic activities of the chemical compounds was estimated by estradiol equivalent (EEQ). EEQi of a particular compound (i) is numerically the product of the compound concentration (Ci) and its oestrogen equivalent factor (EEFi) (Esteban et al. 2014a). EEF or relative potency (Jiang et al. 2012) was obtained from the ratio of median effective concentration (EC_50_) of estradiol (E2) the natural oestrogen, to EC_50_ of the target compound (Jiang et al. 2012). EC_50_ is defined as the concentration of the micropollutant which produces half maximal estrogenic activity. The additivity is obtained by the summation of the individual estrogenic activities viz., EEQ_TOTAL_ = Ʃ(*C*_*i*_ × EEF_i_) (Salgueiro-González et al. 2015).

### Statistical analysis of data

The statistical analyses were performed using IBM SPSS Version 24 (2016) or Microsoft Excel 2010. Level of statistical significance was *p* ˂ 0.05.

## Results

### Physicochemical analysis

Analysis of variance (ANOVA) and Pearson’s correlation coefficient was carried out from the data on physicochemical parameters (Table 2). The range of pH recorded along the river course was 6.4–13.1 in 2016 while the range was 6.1–8.8 in 2017. The average pH among the sampling points was highest at LD (a weir; an impacted site) (Tables 1 and 2) in both years. The pH was not significantly different among the sampling sites in both years. The temperature range was 11.7–19.8 °C in 2016 and was 14.1–19.3 °C in 2017. But the temperatures were significantly different among the sampling points in 2016 unlike in 2017. There appeared to be a progressive increase in turbidity from 24.1 NTU upstream to 168.0 NTU downstream in 2016 and 55.0–371.3 NTU in 2017. The difference in average turbidities among the sampling points in 2016 was statistically significant but not so in 2017. Also in the winter of 2016 but not in 2017, turbidity correlated positively and significantly too with the pH and temperature. Total dissolved solids (TDS) ranged 23.1–317 mg/L in 2016 and 37.0–547.0 in 2017, progressively increasing downstream. When the average TDS values among the sampling points were compared, the differences were significant for both years. Like turbidity, TDS was significantly affected by pH and water temperature (*r* ˃ 0.5). Electrical conductivity (EC) is a useful indicator of salinity or total salt content of the aqueous medium. EC values ranged 32.0–615.0 μS/cm in 2016 and ranged 75–1098 μS/cm in 2017.

**Table 2:** Physicochemical parameters of River Buffalo in winter periods of 2016 and 2017

	Winter year		2016		2017	
Parameters	Sample sites	Sampling number/year	Mean ± SD	*P* value	Mean ± SD	*P* value
pH	MD RD KW ZW LD BD	3 3 3 3 3 3	6.7 ± 0.2 6.8 ± 0.2 6.8 ± 0.7 8.7 ± 0.5 9.8 ± 2.9 9.3 ± 2.8	0.1	6.5 ± 0.4 6.7 ± 0.5 7.4 ± 0.7 7.4 ± 1.2 7.6 ± 1.3 7.5 ± 0.9	0.6
Temperature (°C)	MD RD KW ZW LD BD	3 3 3 3 3 3	13 ± 1.9 15.3 ± 0.9 14.4 ± 1.1 14.9 ± 1.5 17.5 ± 1.4 17.9 ± 1.6	0.01	11.3 ± 2.9 13.0 ± 2.7 11.2 ± 2.5 13.3 ± 3.2 12.6 ± 3.3 15.6 ± 4.1	0.6
Turbidity (NTU)	MD RD KW ZW LD BD	3 3 3 3 3 3	26.7 ± 4.2 54.7 ± 15.1 71.1 ± 56.6 132.6 ± 36.7 146.1 ± 22.5 84.3 ± 32.1	0.01	69.1 ± 16.3 113.3 ± 18.2 156.7 ± 46.7 227.4 ± 126.8 218.0 ± 94.2 192.7 ± 41.8	0.1
Total dissolved solids (mg/L)	MD RD KW ZW LD BD	3 3 3 3 3 3	32.1 ± 9.3 109.2 ± 30.3 78.2 ± 59.4 210.1 ± 66.5 256.0 ± 53.2 180.0 ± 88.1	0.003	39.0 ± 1.7 121.0 ± 137.8 242.7 ± 9.2 328.0 ± 97.0 303.7 ± 216.6 301.3 ± 9.2	0.04
Electrical conductivity (μS/cm)	MD RD KW ZW LD BD	3 3 3 3 3 3	40.7 ± 14.1 152.5 ± 44.0 126.3 ± 103.4 272.1 ± 100.3 402.6 ± 184.9 261.2 ± 133.8	0.02	71.8 ± 10.2 179.3 ± 165.9 405.2 ± 131.2 660.4 ± 199.7 612.7 ± 433.5 590.1 ± 36.4	0.02

Electrical conductivity differed significantly among the sampling points in both years and correlated positively with pH, temperature, TDS and turbidity in 2016, whereas it correlated positively only with turbidity and TDS in 2017.

### Occurrences of 4-tert-octylphenol and triclosan in the river water

#### Method validation

The matrix effect was evaluated for both analytes by preparing a calibration curve with the standards in an extract of the sample water as well as in ultra-pure water. The results showed that the effects of the matrix on the signal were insignificant after sample clean-up using SPE. Analysis of blank carried out did not suggest the presence of external contamination. The efficiency and reproducibility of the method for each analyte were also investigated (Table 3). Samples of river water as well as the Milli-Q ultra-pure water (1 L each) in six replicates were spiked with 2 μg/L of each analyte and was extracted and quantified. The recovery was expressed as % recovery = (A – B/C) × 100 where A = measured concentration in the spiked sample; B = measured concentration in the unspiked sample; C = spike concentration. The precisions were determined and expressed as % relative standard deviation (RSD) which were found to be considerably lower (RSD ≤ 20%) than the maximum value required by SANCO guide (European Commission, 2012). Limits of detection (LOD) and quantitation (LOQ) were calculated by using the standard error of means of the calibration data divided by the slope based on signal to noise ratios of 3.3 and 10 respectively. The results of the method validation study (Table 3) showed that the method was suitable for the analysis of both compounds in the environmental waters and appeared to agree with previous work (Martinez and Peñuela 2013). Sample analyses showed that the two compounds were present in the river water with TCS more frequently present than 4-tOP. Upstream at both MD and RD, the occurrence was either nil or minimal (Fig. 1). The presence of either or both pollutants was recorded in four of the sampling points namely, KW, ZW, LD and BD (Fig. 1). These sites were impacted especially by human-related activities (Table 1).

**Table 3:** Retention time (RT), limits of detection (LOD) and quantification (LOQ), recovery rate (accuracy), precision (% RSD), regression model and regression coefficient in ultra-pure water spiked with standard analytes solution

	RT (mins)	LOD (ng/mL)	LOQ (ng/mL)	Recovery rate (%) *n* = 6	% RSD	Regression model	Regression coefficient (*r*^2^)
4-tert-Octylphenol	9.3	0.30	0.92	100.8 ± 9.2	8.31	*y* = 7E+07x + 5E+06	0.9996
Triclosan	12.7	0.58	1.75	96.7 ± 0.93	8.79	*y* = 3E+07x-2E+06	0.9984

*RSD*, relative standard deviation

**Fig. 3:**
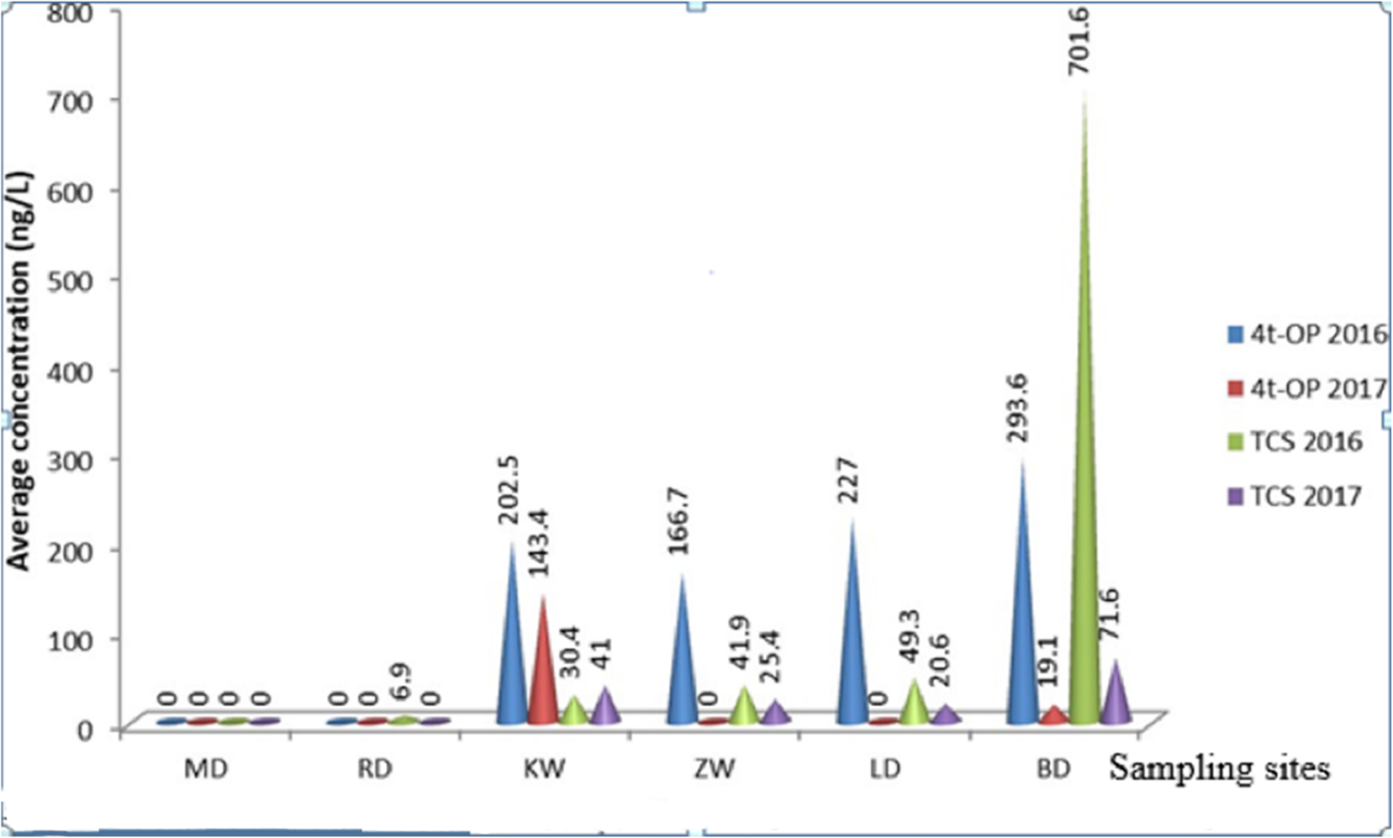
Average concentrations of TCS and 4-tOP in water by sampling sites

Expectedly, the concentrations of the two analytes increased downstream (Fig. 3). The highest concentrations recorded during the periods of this investigation was 755 ng/L for 4-tOP and 1264.2 ng/L for TCS both occurred at BD, downstream of the river. Generally, the concentrations of the two chemical compounds recorded in 2017 were low when compared with 2016 (Fig. 3). On average, a higher amount of TCS was generated in the winter of 2016 than of 2017, whereas higher 4-tOP concentration was generated in 2017 than in 2016. Notwithstanding the higher variation of TCS concentration in the river water, its median concentration was less than that of 4-tOP (Table 4) suggesting that the actual level in the river water of 4-tOP might be higher than that of TCS. What was significant when these results were compared with the literature values (Table 4) was the non-detection of the micropollutants in some samples of the river water regardless of the comparably low LOD and LOQ (Table 3).

**Table 4:** Estimation of estrogenicity index following exposure to 4-tert-Octylphenol and Triclosan for a given period

	4-tert-Octylphenol			Triclosan		
Species	Algae	Daphnia	Rainbow trout	Algae	Daphnia	Rainbow trout
Endpoint	NOEC	LC_50_	NOEC	EC_50_	EC_50_	EC_50_
Test duration	96 h	48 h	96 h	96 h	48 h	96 h
Concentration (ng/L)	1000^1^	620^2^	77^3^	1400^5^	390,000^5^	260,000^5^
Assessment factor^4^	10	1000	10	1000	1000	1000
PNEC (μg/L)	100	0.62	7.7	1.4	390	260
MEC (μg/L)	0.76	0.76	0.76	1.264	1.264	1.264
HQ	0.008	1.23	0.1	0.9	0.003	0.005
Average concentration (ng/L)	143.2			701.6		
EEF^⁎^	1.4 × 10^−4^			1.3 × 10^−2^		
EEQ (ng/L)	0.02			9.1		
EEQ_TOTAL_ (ng/L)	9.12					

*HQ*, hazard quotient; *MEC*, maximum measured environmental concentration; *LC*_*50*_, median lethal concentration; *NOEC*, no observed effect concentration; *PNEC*, predicted no effect concentration; ^**1**^**(**Laboratories Inc. 1984); ^2^(Marcial et al. 2003); ^3^ (IUCLID 2000). ^*4*^ (European Commission 2003; Gros et al. 2010)*;* ^5^(Orvos et al. 2002). *Calculated from median effective concentrations EC_50_ for E2 = 18.3 ng/L; for 4-tOP = 1.33 × 10^5^ ng/L (Yang et al. 2013), for TCS = 1.4 ng/L (Selvaraj et al. 2014). *EEF*, oestrogen equivalent factor; *EEQ*, estradiol equivalent

### Ecological risk assessment

The extent of ecological risk on the pollutant-sensitive aquatic organisms was determined by calculating HQ. Aquatic chronic NOEC (no observed effect concentration) values of 4-tOP and TCS from literature were used to calculate PNECs (predicted no effect concentrations) according to European Commission Technical Guidance Document (European Commission 2003). A “worst-case scenario” was adopted by using maximum environmental concentrations (MEC) in the calculation of HQ (Table 4; Zhao et al. 2010).

An estimate of estrogenicity index as estradiol equivalent (EEQ) (Esteban et al. 2014b) for each pollutant was also made (Table 4). Exposure of the Daphnia to 4-tOP at the maximum concentration recorded in the river was potentially hazardous (HQ ˃ 1) to the organism in contrast to nil or low risk to the algae and fish (HQ ˂ 1) (Table 4), whereas the maximum concentration of TCS in the river water could only pose a low risk (HQ ˂ 1) to algae and near-zero risk (H ≈ 0) to Daphnia and fish (Table 4; Blair et al. 2013). Estimation of the individual and joint contributions to the river’s estrogenicity measured as EEQ showed that TCS may have contributed higher estrogenicity than 4-tOP to the river (Table 4).

## Discussion

The range of concentrations of the two chemical compounds suggested that the two pollutants were not always present in the river water. They could have been partly removed by the water treatment plants located along the course of the river and possibly in addition to (bio)degradation through biological or/and by physical process (Rajendran et al. 2017). The average concentrations of both compounds were highest in 2016 winter, brought about by certain anthropogenic activities which were non-existent in 2017. For example, during the reconnaissance visits in 2016 people of some faith were seen bathing at the banks of the river at BD and LD. The bathing soaps might contain TCS which could increase its concentration in the water. Similarly, refuses of various sorts were found scattered close to the river bank at KW which could be a source of 4-tOP release into the river water. Both scenarios which were conspicuously absent during subsequent visits in 2017 were expected to influence the results of this study. However, the average concentrations of the two chemical compounds in the river water are comparable with the reported values from around the world (Table 5) including those of Peng et al. (2008).

**Table 5:** Concentrations of 4-tOP and TCS in freshwater in comparison with the literature values

Analytes	Frequency of detection (*n* = 36)	Range (ng/L)	Median (ng/L)	Literature values (ng/L)	References
4-tert-Octylphenol	0.3	nd-755	88.1	2.0 601 66.0–474.2 3.1–96.4	Wu et al. 2013 Bergman et al. 2013 Yang et al. 2013 Jiang et al. 2012
Triclosan	0.4	nd-1264.2	82.1	13 62–245 4.3–10.4 1430 2300	Perez et al. 2013 Wang et al. 2013 Shen et al. 2012 Loraine and Pettigrove 2006 Kolpin et al. 2002

*nd*, not detected

The pH of the river water ranged from low acidity upstream to moderately alkaline downstream during the two winter periods (Chigor et al. (2013). The acidic pH of the water consistently recorded at the upper reaches of the river catchment, MD and RD could not have been attributed to human activities per se but might as well be due to organic matter decomposition from the riparian vegetation that dotted these areas (Winterbourn and Collier 1987). The two sites were near-pristine, devoid of anthropogenic activities, thus explaining the near-zero detection of these chemicals in the water sampled from these sites. In the study, the pH of the water could not have played a significant role in deciding the concentration of the chemicals at each sampling sites because the pH values were not significantly different among the sites. Although river waters generally are alkaline or sufficiently above neutrality (Stasinakis et al. 2012; Ishaq and Khan 2013), the pH ranges recorded along the course of the Buffalo River could be injurious to certain aquatic lives (Alabaster and Lloyd 1980) whose activities are expected to contribute to the reduction of these chemicals in water by way of biodegradation (Reis and Sakakibara 2012; Lee and Chu 2015). Loss of both compounds through hydrolysis at the recorded pH range appears insignificant given their lipophilic nature (Singer et al. 2002; US EPA 2008). This could lead to their persistence in the water and to bioaccumulation by the aquatic flora and fauna (Houtman et al. 2004; Capdevielle et al. 2008; Coogan and La Point 2008) putting humans subsisting on the aquatic resources such as fish and water for domestic use, at the risk of the diseases associated with estrogenic endocrine disruptors (De Coster and van Larebeke 2012). At the pH recorded in the lower reaches of the river viz.; ZW, RD and BD, humans could also be exposed to the toxicity of the degradation product 2, 4-dichlorophenol possibly carried out on TCS by certain aquatic flora (Taştana and Dönmeza 2015). The degradation product is thought to be more toxic than its parent compound being responsible for the parent’s pro-oxidant activity (Gou et al. 2014).

Relatively low water temperatures that were recorded at the sampling sites like MD, KW and ZW were afforded by the riparian vegetation present at these areas which provided shade and consequently reduced/altered the ambient temperatures. This condition becomes quantitatively significant on the river body that is directly open to solar radiation, especially at BD. Given the favourable alkaline pH of water at this site for photolysis (Huang et al. 2013), the two chemical compounds were capable of undergoing solar transformation leaving octylcathecol from 4-tOP (Huang et al. 2013) and chlorophenols (Canosa et al. 2005) from TCS in the water, both metabolites being more toxic than their respective parent compounds (Gurban et al. 2015). Turbidity measures the cloudiness of the water occasioned by the presence of suspended particles of silt, clay, waste effluents and other particulate materials possibly originating from natural sources such as peaty waters from upland areas as well as from human activities. In all the samples, turbidity was found to be directly related to pH, EC and TDS as also reported by Singh and Sharma 2016. Turbidity reduces light penetration into the water and hence denying plankton and other benthic organisms access to sunlight leading to a deficit in photosynthetic activity and consequently threatening the survival of the higher members of the trophic levels (Sharma et al. 2016). The increased turbidity may mean an increase in available particulates for sorption of the pollutants (pK_OW_ > 4) (Reiss et al. 2002; Dhillon et al. 2015) or may mean reduced photo-degradation of the chemical compounds. Both processes ultimately lead to a reduction of the chemicals or their toxic metabolites in the water consequently reducing human exposure and hence reduced toxicity to humans. Sampling sites in 2016 might have differed by the turbidity level of the water given the level of statistical significance (*p* = 0.01). We observed this might be due to increased anthropogenic activities like refuse dumping among the sites when compared with 2017. Total dissolved solids (TDS) in water compose mainly of carbonates, bicarbonates, chlorides, phosphates and nitrates of calcium, magnesium, sodium, potassium and manganese; organic matter, salt and other particles (Trivedy and Goel 1986) which may increase the salinity of the water and may render it unfit for agricultural and domestic purposes. An inverse relationship between the growth of aquatic taxa and TDS exposure was reported by Olson and Hawkins (2017). They concluded that high TDS exposure resulted in the low growth rate of the invertebrates that were tolerable to low TDS. The highest TDS ever recorded and also for this study along the river course was less than 500 mg/L, the maximum EPA (2002) recommended value. The increased TDS could lead to eutrophication especially at Laing and Bridle Drift Dams that could lead to increased turbidity and reduced concentration in water (Water Research Commission 1996) and possibly reduced human exposure.

The estimated total estrogenicity (EEQ_TOTAL_) of the river water occasioned by the two chemical compounds is comparable with the literature values from around the world but could be rated high when compared with the South African drinking water (Table 6).

**Table 6:** Estrogenic activities in some surface waters around the world

EEQ (ng/L)	Source water	References
0.2–2.4	River system (6 Nos.), China	Ramaswamy et al. 2011
0.2–9.4	Yellow River, China	Wang et al. 2012
0.2–324	The Pearl River, China	Zhao et al. 2011
0.6–2.5	Drinking water, South Africa	Aneck-Hahn et al. 2009
0.02–1.9	Youngsan River, Korea	Oh et al. 2006
0.7–4.0	Tokyo Bay, Japan	Hashimoto et al. 2005
2.8–81.4	Flemish Rivers, France	Witters et al. 2001

### Ecological risk assessment

HQ, an index of ecological risk of ED (Chen et al. 2014), is defined as the ratio of the environmental concentration of the chemical stressor to the predicted no effect concentration (PNEC) (Ramaswamy et al. 2011). PNEC is calculated either from acute toxicity (LC_50_ or EC_50_) or chronic toxicity values (NOEC) with an assessment factor of either 1000 or 10 respectively (Selvaraj et al. 2014). A compound is of low risk when HQ value is ˂ 1 while higher values (HQ ˃ 1) imply greater ecological risk. For 4-tOP, the sensitivity was according to Daphnia ˃ fish ˃ algae in agreement with the previously reported work (Di Paolo et al. 2016) while sensitivity to TCS varied according to the trend algae > daphnids = fish. Daphnia or water flea is a freshwater planktonic crustacean often found pelagic, predatorily by fish while algae are primary producers in the ecosystem and their photosynthetic machinery has been reported to be adversely affected by TCS (Eriksson et al. 2015). The loss of the photosynthetic ability of the algae following exposure to TCS may lead to acidification of the river water (Xin et al. 2019) as the result of CO_2_ build-up. TCS induced concentration-dependent genotoxicity has also been reported in certain species of algae namely *Asterococcus superbus* and *Chlamydomonas reinhardtii* (Xin et al. 2019) which abound in freshwater such as the Buffalo River. These instances are capable of producing a negative shift in the balance of the ecosystem. HQ as high as 28 has been reported from river waters in China (Singh and Sharma 2016) much higher than from this study. Concentration addition (CA) based on the concept of additivity, developed by Loewe and Muischnek (1926) has been used in assessing the mixture effects of ED (Yang et al. 2014). Toxicity arising from mixture effects may be additive, synergistic or antagonistic depending on whether the effects correspond to the sum of each component’s effect, higher than the summed up effect of the individual substances, or the effect is below the sum of individual effects. Concept of concentration addition was invoked in this study because both 4-tOP and TCS target ER. EEQ estimates estrogenic contribution to the river by the target ED which could be obtained either from bioassays or by calculation from chemical analysis (Jiang et al. 2012) both of which have been reported to be correlated positively as well as complementary (Ihara et al. 2015). Direct EFF value for TCS was not obtainable from the literature including US EPA for reason unknown to us at this time. Consequently, EFF for TCS and for 4-tOP were derived from the EC_50_ values from the literature. In the case of multiple values for an EC_50_, the least was selected for each compound. Estrogenic compounds present in water may affect the aquatic organisms by disrupting their hormonal homeostasis (Manfo et al. 2014). Reports on male fish feminization and loss of the fish population were found to be strongly linked to exposure to natural or synthetic estrogens at concentrations as low as a few ng/L. Laboratory and field investigations have shown that EEQ values in the range 2.7–13.6 ng/L can increase plasma VTG levels in fish while values ranging between 13.6 and 136.2 ng/L can lead to a decrease of testicular growth (Pojana et al. 2004). The estimate of total estrogenicity (Table 5) notwithstanding the low HQ might have shown that both compounds were capable of causing an imbalance in the aquatic ecosystem at the recorded concentrations. The amount of estrogenicity contributed by TCS in this study could be considered as high enough to cause increased plasma VTG in the fish when placed side by side other laboratory results (Table 6). TCS inhibits estradiol (E2) sulphonation in sheep placenta (James et al. 2010) which may delay E2 excretion. Inhibition of human aromatase or cytochrome P450 19A1 activity in human choriocarcinoma trophoblastic cells (JEG-3) by TCS has also been reported (Wu et al. 2013) possibly resulting in diminished estradiol production (Kjeldsen et al. 2013). This may be the anti-estrogenic potency of TCS which is worthy of further studies. Exposure to 4-tOP has been shown to produce adverse effects on reproduction, alteration of the sex ratio and development (Othman et al. 2012). An example of the adverse effect of estrogenic compounds including alkylphenol (4-tOP) on reproduction dated back in 1978 which produced feminization of fish in the English rivers reported by Sumpter and Johnson (2008). The report of the study in Ontario, Canada on an experimental lake suggested that the intersex effect of ED on fathead minnow fish caused a progressive reduction in the fish population (Kidd et al. 2007). 4-tOP stimulates the expression of cathepsins in human breast cancer cells and xenografted breast tumours of a mouse model via an oestrogen receptor–mediated signalling pathway (Lee and Choi 2013). Exposure of males to either 4-tOP or TCS produced idiopathic infertility (Chen et al. 2013).

### Conclusion and future directions

Save the upper reaches, the river is moderately impacted during the wintertime. The sampling points differed by physicochemical qualities of the water samples which presumably affected the distribution of the pollutants. The average concentrations of the two ED from the river water were within permissible limits of human consumption but outside the safety net for lower organisms. An issue of underestimation of the two ED might need further studies to include those sorbed on the river sediments. However, this work may have produced a baseline for further study of these two chemical compounds in the river water. Extensive work covering all seasons needs be undertaken to make an absolute pronouncement about the levels of these chemical compounds in the river. Similarly, methods of assessing ecological risk should have been expanded to include a bioassay for the determination of overall river water estrogenicity (Manickum and John 2014). Following these deficiencies, the data from this work could not have passed for absolute information about the 4-tOP and TCS removal efficiency of the wastewater treatment plants located along the course of the river Buffalo.

## Data Availability

Available.
